# Case 1/2018 - Preponderant Left Ventricular Restrictive Syndrome in a 28-Year-Old Woman

**DOI:** 10.5935/abc.20170186

**Published:** 2018-01

**Authors:** Edmar Atik, Danielle Haddad Syllos Dezen

**Affiliations:** Hospital Sírio Libanês, São Paulo, SP - Brazil

**Keywords:** Cardiomyopathy, Restrictive, Atrial Fibrillation, Electroshock, Ventricular Dysfunction Left, Hypertension, Pulmonary

**Clinical data**: Increase in size of the left atrium known since 8 years of age, when she was labeled as carrier of idiopathic left ventricular restrictive syndrome. Since then, there was a progressive increase of this cavity with the appearance of fatigue to the efforts and atrial fibrillation five months ago, reversed in the occasion with electric shock and amiodarone. Despite a high dose of this drug, 400 mg/day, heart rate remained elevated, around 100 bpm, having developed thyroid dysfunction with TSH elevation and hormone diminution. The arrhythmia reappeared two months later and led to new clinical research.

Physical Exam: eupneic, acyanotic, normal pulses, HR = 96 bpm, BP = 100x70 mmHg.

Venous impulses in the neck. In the precordium, there were no impulses, normal heart sounds, no audible murmurs. Third sound sometimes inconstant. Clean lungs. Abdomen unchanged.

## Additional tests

**Figure 1:**
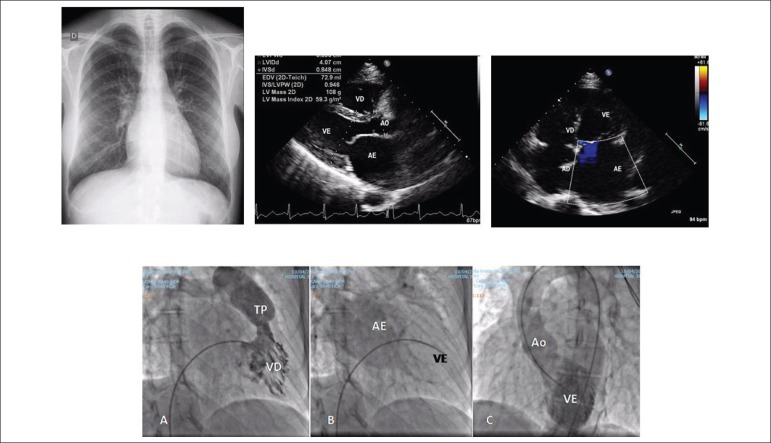
Chest X-ray in AP emphasizes normal cardiac area with a clear increase of the left atrium size in a double contour in the lower right arch and slightly congested pulmonary vascular weave in the upper fields. The echocardiographic images highlight the left atrial enlargement in longitudinal and 4-chamber projection. Angiocardiograms below show right ventricular hypertrophy (A), left atrial emptying delay (B) compatible with left ventricular restrictive syndrome and this cavity with smooth and normal-sized internal borders (C).

**Chest X-ray**: normal cardiac area with a clear increase of the left atrium in a double atrial contour and a more prominent pulmonary vascular weave in the upper fields (Figure 1).

**Electrocardiogram:** atrial flutter and right bundle branch conduction disorder with rr1 morphology in V1. AQRS = +70º AT = + 110º.

**Echocardiogram:** exclusive increase of the left atrium. Ao = 28, LA = 53, RV = 22, LV = 40, septum = 8, posterior wall = 10, LVEF = 68%, SPRV = 53 mmHg. Enlarged superior vena cava without ventricular dyskinesias (Figure 1).

**Magnetic nuclear resonance**: no fibrosis in late enhancement.

**Cardiac catheterization:** showed signs of restriction to left ventricular filling with discrete pulmonary hypertension. LA = 12, RV = 35/10, PA = 35/20-28, Wedge = 20, LV = 80/5-18, Ao = 80/50-64, CO = 3.1 l/m, PVR = 2.58 W, SVR = 16.75 W Angiography clearly emphasized the left atrial emptying delay, right ventricular hypertrophy, and the left ventricular endocardial smooth wall (Figure 1).

**Endomyocardial biopsy of the RV septum**: Five fragments of the right ventricle myocardium with elastic consistency and brownish, measuring 4x3x1 mm, showed moderate and diffuse hypertrophy in cardiomyocytes, moderate and focal interstitial myocardial fibrosis, absence of inflammatory infiltrate, absence of amyloid protein deposition by the Red Congo histochemical method, negative histochemical investigation of glycogen and neutral mucopolysaccharides, and acids by the periodic acid method of Schiff, with and without diastase.

**Clinical diagnosis**: idiopathic restrictive syndrome with isolated increase of the left atrium size with atrial flutter and pulmonary hypertension, in addition to hypothyroidism due to the use of high dose of amiodarone.

**Clinical reasoning**: In an initially asymptomatic patient with an even slight increase in the left atrium, without other commemorative ones, the diagnosis of restrictive left ventricle syndrome becomes imperative. The progressive increase of this cavity further accentuates this diagnosis, even more with the advent of pulmonary congestive symptoms expressed by dyspnea, physical fatigue and enlargement of the pulmonary vascular network, in addition to the appearance of atrial flutter. Restrictive syndromes are classified into myocardial, non-infiltrative idiopathic, infiltrative amyloidosis type, sarcoidosis, Gaucher and Huler disease; storage diseases such as hemochromatosis, Fabry disease and glycogen storage. We can also mention the endomyocardial ones with fibrosis, eosinophilic syndrome, the carcinoid, those by irradiation, of malignant tumors and by anthracycline toxicity.

**Differential diagnosis**: symptoms of fatigue, atrial size increase, pulmonary arterial hypertension and ventricular diastolic dysfunction, assure the diagnosis of restrictive syndrome. Therefore, the cause should immediately be sought from those exposed above, through diagnostic imaging, allied to cardiac biopsy. Treatment depends on the cause, corticosteroids on sarcoidosis, chelation on hemochromatosis.

**Conduct**: having found mild pulmonary arterial hypertension, there was an indication for the use of metoprolol 100 mg/day adrenergic beta-blocker in order to improve the ventricular filling and to decrease the heart rate after the obligatory suspension of amiodarone. Atrial flutter ablation was also indicated to improve ventricular filling and to prevent the left atrial enlargement. Routine clinical follow-up aims to preserve the current condition in order to postpone cardiac transplantation.

**Comments**: The main characteristic of the restrictive syndrome is diastolic filling difficulty, with normal ventricular volume and more rigid ventricular walls despite normal thickness, and also with preservation of systolic function. Therefore, it provokes proportional increase of the left atrium, congestion and pulmonary arterial hypertension. Therapeutic options are rare and ineffective and cardiac transplantation should be considered in advanced phases.^1,2^ Left atrial septum decompression by atriosseptostomy has been considered in order to reduce congestion and pulmonary hypertension, allowing to postpose the indication for transplantation.^2^ It is the least common cardiomyopathy of all types. The genetic spectrum points to mutations in sarcomeric genes in half of the cases.^1^ Evolutionarily, sudden death occurs in 80%, heart failure in 15%, infective endocarditis in 5%, predominantly below 20 years of age.
